# The functionality of a therapeutic antibody candidate restored by a single mutation from proline to threonine in the variable region

**DOI:** 10.1080/21645515.2023.2279867

**Published:** 2023-11-27

**Authors:** Marie Hautiere, Irene Maffucci, Narciso Costa, Amaury Herbet, Sosthene Essono, Séverine Padiolleau-Lefevre, Didier Boquet

**Affiliations:** a Département Médicaments et Technologies pour la Santé (DMTS), SPI, Université Paris-Saclay, CEA, Gif-sur-Yvette, France; b Centre de Recherche de Royallieu, CNRS UMR 7025, Génie Enzymatique et Cellulaire, Compiègne Cedex, France; c Centre de Recherche de Royallieu, Sorbonne Universités, Université de Technologie de Compiègne, Génie Enzymatique et Cellulaire, Compiègne Cedex, France; d Medical Biotechnology Engineering LLC, Malden, MA, USA

**Keywords:** Monoclonal antibody, chimerization, proline, *in silico modelling*

## Abstract

mAbs play an essential role in the therapeutic arsenal. Our laboratory has patented the Rendomab-B49 mAb targeting the endothelin B receptor (ET_B_). This G protein-coupled receptor plays a driving role in the progression of numerous cancers. We chimerized our mAb (xiRB49) to evaluate its preclinical therapeutic efficacy in different ET_B_^+^ tumor models with an antibody drug conjugate approach. As previously reported, the chimerization process of an antibody can alter its functionality. In this article, we present the chimerization of RB49. xiRB49 purified by Protein A remained perfectly soluble and did not aggregate, but it lost all its ability to recognize ET_B_. A detailed analysis of its variable region using IMGT tools allowed us to identify an unusual proline at position 125. *In silico* mAb modeling and *in vitro* experiments were performed for a better understanding of xiRB49 structure-function relationships. Our results show that the proline in position 125 on the heavy chain alters the xiRB49 CDR3 light chain conformation and its mutation to threonine allows complete functional recovery.

## Introduction

The endothelin axis, composed of two G-protein-coupled receptors, ET_A_ and ET_B_, and three ligands, ET-1, −2, and −3, is involved in many physiological functions.^1^ The deregulation of this axis is associated with a large variety of diseases.^2,3^ In a tumor context, several studies have demonstrated that the ET_A_ and ET_B_ signaling pathways are involved in several hallmarks of tumor progression.^2,4,5^ Thus, endothelin receptors have become a major target for cancer treatment as illustrated by numerous clinical trials (NCT04205227, NCT04158635, NCT05072106).^6–8^

We have published studies on our antibodies named Rendomab B1, B4, B49 and A63 targeting ET_A_ or ET_B_, which display remarkable properties.^9–12^ Concerning ET_B_, we have shown that Rendomab B1 is one of the most effective ET-1 antagonists.^9^ However, in a tumor microenvironment rich in ET-1, it is more appropriate to consider an antibody, such as the patented Rendomab B49 (RB49), whose binding will not be impaired by the high endothelin concentrations,^13^ specifically, to target ET_B_^+^ tumor cells in an ET-rich microenvironment such as in glioblastoma, melanoma and myeloma.^11,14^ In this short report, we describe the essential step of RB49 chimerization with human IgG1-Kappa isotypes (xiRB49). We assessed physicochemical parameters of xiRB49, namely its hydrodynamic radius, molecular weight, temperature curve, inflection temperature, specificity and affinity. We report the complete loss of functionality of xiRB49, despite its solubility and absence of aggregation, while its recombinant murine version was fully active. We detected an unusual proline at position 125 of the heavy chain variable region using IMGT/V-QUEST and IMGT alignment of mouse IGHJ alleles.^15^ So, we investigated RB49, xiRB49, and xiRB49-P125T, a mutant with a classical threonine at position 125, by *in silico* approaches combined with *in vitro* experiments. These results highlighted that the proline at position 125 in the heavy chain of xiRB49 alters the relative conformation of complementarity-determining regions (CDRs), while its mutation to threonine produces a fully functional xiRB49-P125T.

## Results

### xiRB49 expression and functionality

**Figure 1.**
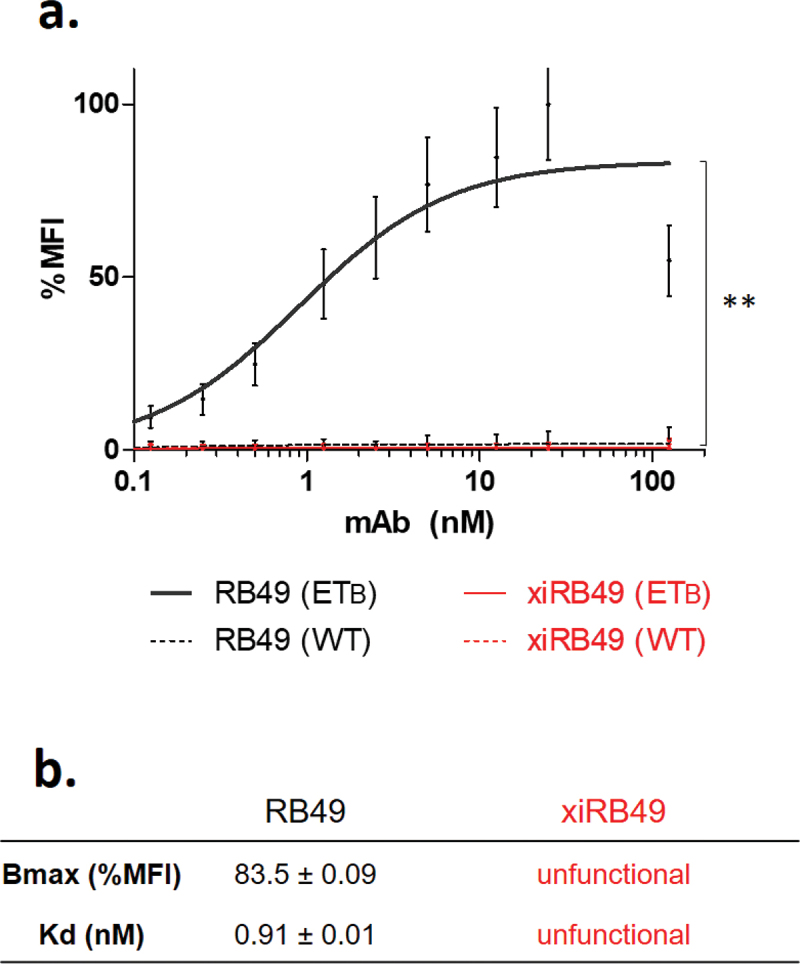
Antibody functionality assay. (a) FACS binding curves of RB49 (black) and xiRB49 (red). MAb specificity was assessed with CHO-ET_B_ (solid line) and CHO-WT (dotted line). (b) Table of apparent Kd (nM) and Bmax (% MFI) for RB49 and xiRB49. Data are presented as mean ± SD.

xiRB49 was expressed in ExpiCHO^TM^ cells and purified on a protein A column, suggesting correct refolding of the constant IgG1 domain (Figures S1 and S2a). Moreover, we observed, on SDS PAGE, in non-reducing conditions, a band at 150 kDa and in reduced conditions two bands corresponding to the heavy and light chains 50 kDa and 25 kDa, respectively (Figure S2b). We determined a hydrodynamic radius (Rh) of 5.2 ± 0.4 nm and no aggregation was detected (Figure S2C). Identical results were obtained for our positive control, the chimeric Rendomab A63, xiRA63, already described for theranostic applications to ET_A_^+^ tumors.^12^ We assessed the functionality of xiRB49 and RB49 by their binding to CHO-ET_B_ cells compared to CHO-WT cells (Figure 1). No recognition for ET_B_ was observed with xiRB49. In contrast, RB49 was fully functional with an apparent Kd = 0.91 ± 0.01 nM and a Bmax value (maximal Binding ability of the antibody) of 83.5 ± 0.09% MFI (Mean Fluorescence Intensity). No RB49 cross-reaction was detected with CHO-WT cells, highlighting its specificity.

As RB49 was purified from hybridoma, we decided to produce its recombinant format (reRB49) in ExpiCHO cells to evaluate its functionality. FACS (Fluorescence Activated Cell Sorter) binding curves indicated that reRB49 was fully functional with identical RB49 parameters (Figure S3). This indicates that the origin of the loss of ET_B_ recognition by RB49 variable regions is due to the chimerization process with the human IgG1Kappa isotype.

**Figure 2.**
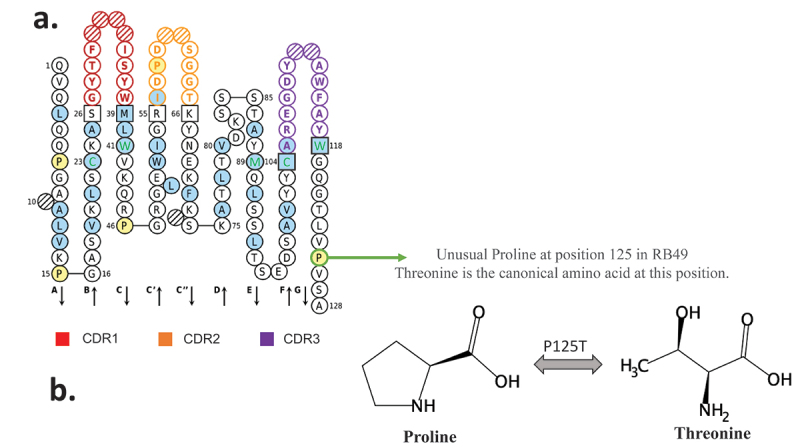
RB49 VH domain IMGT collier de perles representation. (a) All hydrophobic amino acids are in blue and amino acids conserved positions in all immunoglobulins in green. Squares represent the anchor positions which delimit the CDR-IMGT (framed in color, red CDR1, orange CDR2, purple CDR3) and framework IMGT (framed in black) regions. Hatched circles correspond to missing positions according to IMGT unique numbering for variable domain. The direction of the beta strands is represented by black arrows. Unusual proline is circled in green (b) proline and threonine chemical structures are presented.

**Figure 3.**
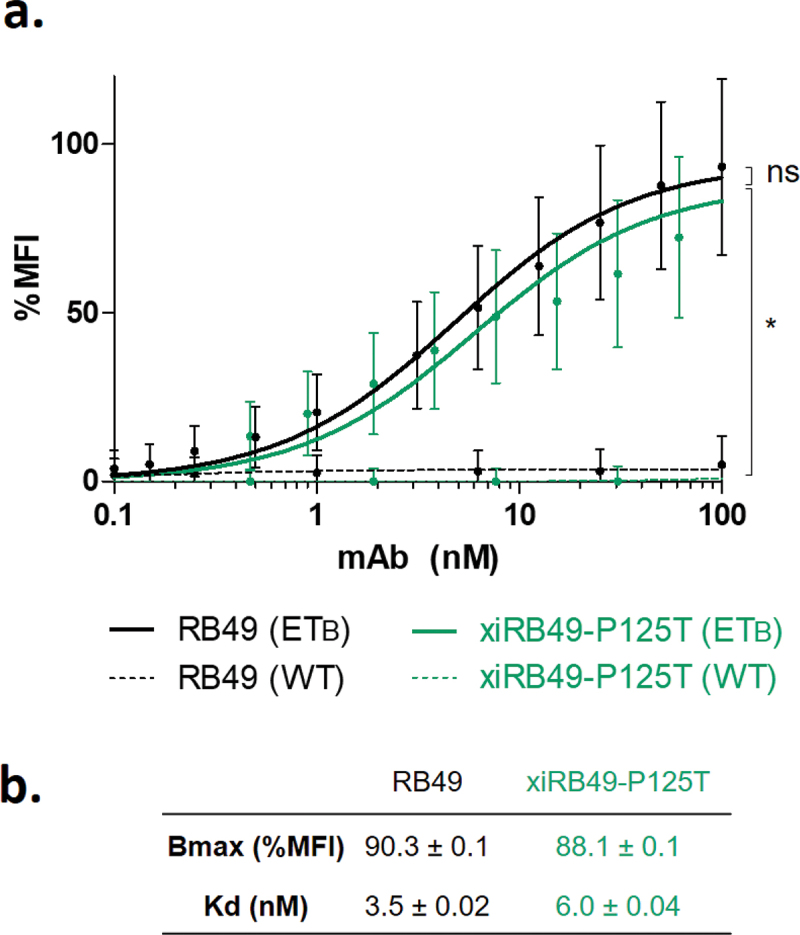
FACS binding curves of RB49 and xiRB49-P125T. (a) RB49 (black) and xiRB49-P125T (green) for CHO-ET_B_ (solid line) and CHO-WT (dashed line). (b) Apparent Kd (nM) and Bmax (% MFI) are indicated in the table. Data are presented as mean ± SD. A two-tailed paired student’s t-test was used for data comparison.

A careful analysis of variable sequences allowed us to identify an unusual proline at position 125. Indeed, no proline is encoded by the mouse genome in the FR4 domain (Figure S4).The RB49 VH domain is represented according to the IMGT rules and numbering. The unusual proline is circled in green (Figure 2). As the canonical amino acid at this position is threonine, we decided to explore the functionality of the xiRB49-P125T mutant. A fully functional xiRB49-P125T was restored, as confirmed by its binding curves, with an apparent affinity similar to that of RB49. As expected, no binding to CHO-WT cells was observed (Figure 3).

### Study of the disruptive role of proline in the RB49 chimerization process

**Figure 4.**
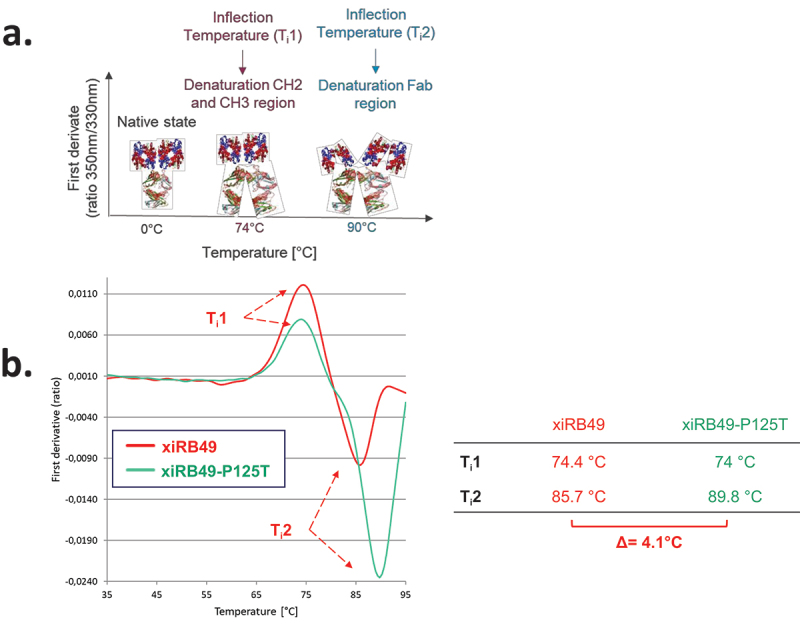
xiRB49 and xiRB49-P125T thermal denaturation curves. (a) Schematic representation of the denatured domain of an antibody as a function of the temperature. (b) First derivative (ratio A350 nm/A330 nm) curves as a function of temperature for xiRB49 (red) and xiRB49-P125T (green). Inflection temperature (T_i_) (table). HC: heavy constant chain; T_i_: inflection temperature.

**Figure 5.**
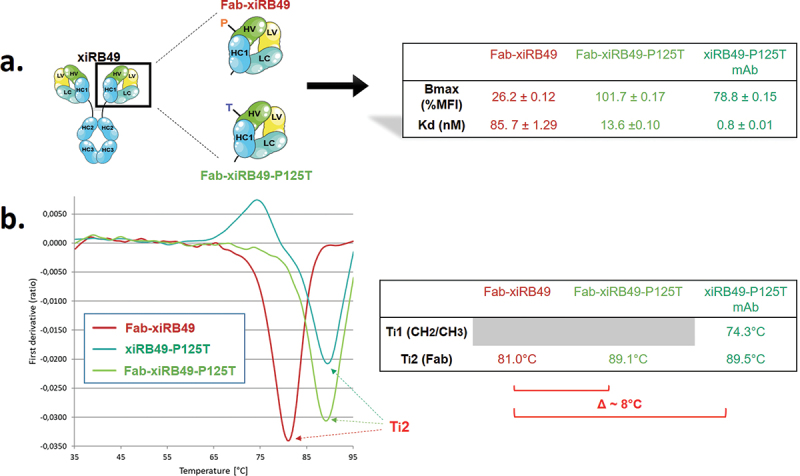
Binding and thermal denaturation experiments with xiRB49 and xiRB49-P125T Fab. (a) Bmax and Kd (table) of the Fab-xiRB49 (red), Fab-xiRB49-P125T (green) and xiRB49-P125T mAb (dark green). (b) First derivative (ratio A350 nm/A330 nm) curves as a function of temperature for Fab-xiRB49 (red), Fab-xiRB49-P125T (green), the xiRB49-P125T mAb (dark green) and their inflection temperature (T_i_) (table).

To understand the influence of proline on the stability of the chimeric RB49, we performed thermal denaturation experiments. We obtained two inflection temperatures T_i_1 and T_i_2 for xiRB49 and the mutant xiRB49-P125T reflecting two transition states in the protein unfolding process (Figure 4a). For T_i_1, reflecting the CH2/CH3 domain denaturation, no difference was observed between xiRB49 T_i_1 = 74.4°C and xiRB49-P125T T_i_1 = 74°C. In contrast, for T_i_2, reflecting Fab denaturation, a difference of 4.1°C was observed between xiRB49 (T_i_2 = 85.7°C) and xiRB49-P125T (T_i_2 = 89.8°C) (Figure 4b). These results underlined that P125 induced structural instability in the chimeric Fab domain. Due to the large difference in amino acid composition between xiRB49 and RB49, we observed that the xiRB49 antibody presents a different profile curve than that of RB49 and their difference in T_i_ is not informative regarding the role of proline in the stability of RB49 (Figure S5). We produced both Fab-xiRB49 and Fab-xiRB49-P125T fragments. Fab-xiRB49-P125T was fully functional and displayed a high affinity (13.6 nM), albeit lower than that of xiRB49-P125T (0.8 nM). Its Bmax value of 101.7% MFI was higher than that of the mAb, 78.8% MFI suggesting that steric hindrance of the full antibody prevents full saturation of the binding sites (Figure 5a). Remarkably, its T_i_2 of 89.1°C agreed with the T_i_2 of 89.5°C of the mAb (Figure 5b). Fab-xiRB49 displayed reduced functionality, with an affinity of 85.7 nM, a low Bmax of 26.2% MFI and a T_i_2 of 81.0°C. The further decrease of 8°C in the T_i_2 of Fab-xiRB49 suggested increased instability in the unmutated chimeric Fab.

### Molecular insights from in silico studies

In order to investigate the relationship between the activity loss of xiRB49 and its structure, we performed accelerated molecular dynamics (aMD) simulations on the RB49, xiRB49 and xiRB49-P125T Fabs, for a total of 10.5 μs of simulation.

**Figure 6.**
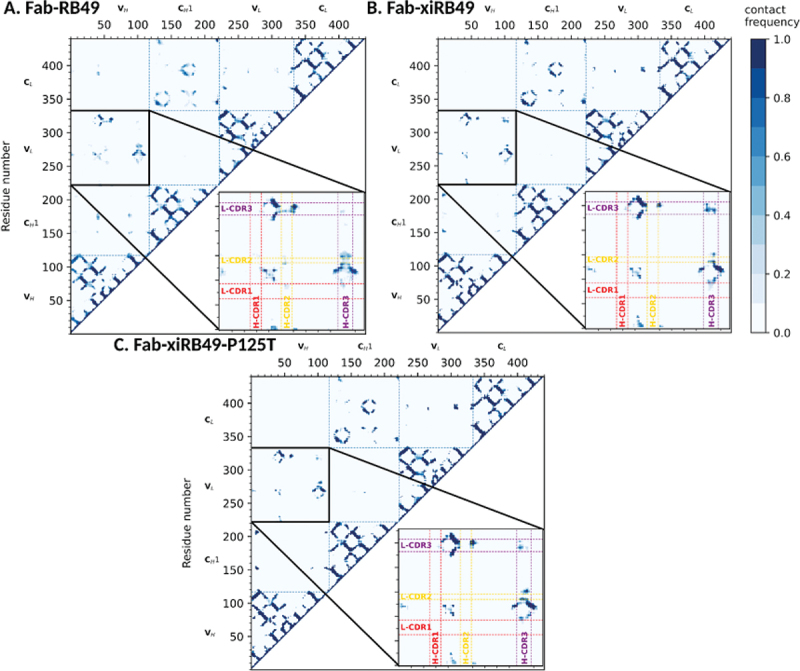
Average contact maps for (a) RB49 Fab, (b) xiRB49 Fab, and (c) xiRB49-P125T Fab. The contact maps were obtained by averaging over the three aMD runs performed for each system and by imposing a Cα distance threshold of 12 Å. The heavy and light chain CDRs are highlighted in red (CDR1), yellow (CDR2), and purple (CDR3). The numbering of amino acids does not correspond to the IMGT numbering, but follows the simulation package sequential numbering (heavy chain variable region: res. 1–118; heavy chain CH1: res. 119–220; light chain variable region: res. 221–332; light chain constant region: res. 333–440).

For each system, average contact maps using a Cα distance threshold of 12 Å (see Materials and Methods section) were computed. Figure 6 shows that, although the contact maps are globally similar, there are some relevant differences in the contacts between the variable regions of the heavy and light chains. In particular, the Fab-xiRB49 heavy and light chains CDR3 (H-CDR3 and L-CDR3, respectively) are in contact during most of the simulation time (Figure 6b), while these contacts are absent during the simulations of Fab-RB49. As for Fab-xiRB49-P125T (Figure 6c), we observed a residual contact between H-CDR3 and L-CDR3. However, this contact did not involve the whole CDR3s as for the Fab-xiRB49 and its contact frequency was lower than that observed in this latter, indicating a less stable contact. This is also visible in Figure S6, showing the probability distributions of the H-CDR3 and L-CDR3 center of mass distances for the three considered systems and in Figure S8, where the solvent accessible CDRs surfaces are showed.

In addition, Fab-RB49 H-CDR3 seemed to be closer to residues framing L-CDR2 for half of the simulation (Figure 6a), while these contacts were absent or less stable for Fab-xiRB49 (Figure 6b). The P125T mutation seems to increase the stability of some of these contacts, suggesting that, thanks to the mutation the Fab-xiRB49-P125T, CDRs assume a conformation closer to that of Fab-RB49 (Figure 6c).

**Figure 7.**
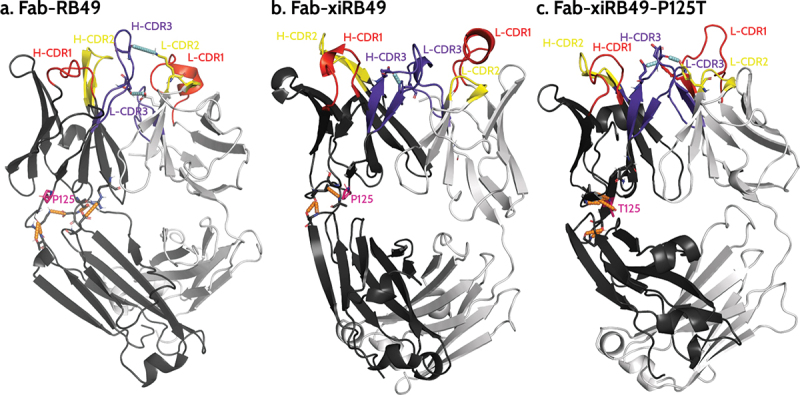
Representative structures of the most populated cluster of (a) Fab-RB49, (b) Fab-xiRB49, and (c) Fab-xiRB49-P125T. The heavy chain and the light chain are represented in dark and light gray, respectively. CDR1, CDR2, and CDR3 are colored red, yellow, and purple, respectively. The residue in position 125 (either proline or threonine) is represented as ball and sticks and colored magenta. The hydrogen bond network within the region between the heavy chain variable and constant regions is indicated as dotted orange lines, while the interactions involving the H- and L-CDRs mentioned in the manuscript are indicated as dotted cyan lines. The indicated hydrogen bonds come from the analysis of the 3 simulations for each system.

We further investigated how chimerization can lead to the observed structural change and, more interestingly, how a single point mutation allowed restoring the affinity for ET_B_. Thus, we performed an analysis of the hydrogen bonds network in the simulations performed on the three considered Fabs (see Materials and Methods section). This analysis highlighted the presence of a network of stable hydrogen bonds (H-bond) involving the heavy chain variable region (where the P125 is located) and the heavy chain constant regions (Figure 7 and Table S1) of Fab-RB49. We observed a highly stable (occupancy >70%) H-bond between D188 and either S96 (in 2 out of the 3 simulations) or R45 (in the third simulation), a stable (occupancy >40%) H-bond between A128 and T131, and a poorly stable (occupancy ~20%) H-bond between S127 and A129 or K130 (Figure 7a, in orange). In addition, for this Fab we observed in 2 out of 3 simulations a stable H-bond (occupancy ~60%) between H-CDR3 E107 and light chain E40, which is immediately after the L-CDR1, and an additional interaction between H-CDR3 D109 and L-CDR2 K56, whose stability depends on the simulation (in cyan in Figure 7a). This, together with globally stabilizing the relative conformation of these regions, might allow proper orientation of H-CDR3, which involves residues C104-W118, ultimately allowing recognition of ET_B_. Conversely, this same analysis performed on the simulations with Fab-xiRB49 (Table S2) showed that the H-bond network surrounding P125 is significantly reduced as compared to Fab-RB49, except for two highly unstable (occupancy ~20%) H-bonds between the heavy chain variable region V13 and the heavy chain constant region S208, only present in one out of the three simulations, and between the heavy chain variable region S127 and the heavy chain constant region A129 (Figure 7b, in orange). Moreover, unlike this latter, for Fab-xiRB49 we observed the presence of a stable H-bond (occupancy >50%, in cyan in Figure 7b) between H-CDR3 (E107) and L-CDR3 (W116), further confirming the proximity of these two regions and the alteration of their relative conformation. Conversely, the P125T mutation in Fab-xiRB49 allows the recovery of some stable interactions in the region structurally close to the mutation (Table S3): a highly stable interaction (occupancy of ~ 70%) between S127 and A129, and an extremely stable interaction (occupancy >90%) involving T125 and A11 and, in one simulation, an additional one between T125 and V13, respectively (Figure 7c, in orange). Moreover, for Fab-xiRB49-P125T, although in two out of the three simulations the H-bond between H-CDR3 E107 and L-CDR3 W116 is still observable, we noticed H-bonds between H-CDR3 and L-CDR2 or L-CDR1 similar to those observed for Fab-RB49, e.g. the interaction between H-CDR3 D109 and L-CDR2 Y55 or K56 and between H-CDR3 E107 and L-CDR1 Y38 (E40 in Fab-RB49) in one simulation (Figure 7c, in cyan), or that between H-CDR3 R106 (E107 in Fab-RB49) and the light chain E40 in the other two simulations. Altogether, this affects the orientation of xiRB49-P125T H-CDR3 and might influence the recovery of the binding affinity for ET_B_.

## Discussion

As widely described in the literature, the processes of chimerization and humanization of an antibody can alter its solubility and functional properties.^16^ Antibodies can be engineered by site-directed mutagenesis to provide properties optimized for theranostic applications, a tedious and antibody-dependent process.^16–18^ MAb chimerization is not always an obvious process. Indeed, chimerization of xiRA63 with the same human IgG1 constant regions, kappa, retained full functionality.^12^ Conversely, xiRB49 completely lost its capacity to bind ET_B_. Interestingly, Fab-xiRB49 presented residual activity with an apparent affinity of 85.69 nM and a reduced Bmax of 26.19% MFI, suggesting that the activity loss of xiRB49 partially comes from the CH2 and CH3 constant domains. However, the amino acid composition analysis of the variable regions revealed an unusual P125 in the heavy chain variable region. It is well known that proline has a strong effect on the secondary structures of proteins,^19–21^ because of proline cis-trans isomerization and because proline cannot form hydrogen bonds with its backbone nitrogen. We checked whether this proline could be encoded in the mouse germlines *IGHJ1*, *IGHJ2*, *IGHJ3*, and *IGHJ4*, which encode framework 4, but no proline was detected. Our reliable cloning strategy for RB49 cDNAs confirms the presence of this P125.^22^ The identical functionality of the reRB49 and the RB49 confirms the reality of proline in position 125. The absence of proline at this position encoded by the immunoglobulin genes suggests that its presence is the result of an affinity maturation process that changes the first A base of the threonine codon (ACN) to the first C base of the proline codon (CCN). A single A-to-C mutation is sufficient and necessary to mutate threonine to proline. Since RB49 is generated by *in vivo* gene immunization in mice, this mutation was retained during clonal selection of B lymphocytes and maturation of RB49 affinity.^23^ The recognition properties of ET_B_ by RB49 are unique in that we have shown that RB49 is able to recognize the full range of ET_B_ conformations presented on the surface of several melanoma cell lines.^11^

To recover a functional xiRB49, we performed a single P125T mutation, since threonine is the canonical amino acid at this position. This allowed both Fab and mAb formats to fully recover their ET_B_ binding properties, indicating that P125 not only does not play an essential role, but is even detrimental for ET_B_ recognition in chimeric RB49. Indeed, the dramatic impact of P125 on RB49 stability and functionality is also highlighted by the inflection temperatures of xiRB49, xiRB49-P125T, Fab-xiRB49 and Fab-xiRB49-P125T. In fact, Fab fragment T_i_2 showed a variation of 8°C between mutated and unmutated fragments.

In order to have a molecular picture explaining the structural role played by P125, we *in silico* modeled the Fab-RB49, Fab-xiRB49 and Fab-xiRB49-P125T, by combining the Antibody modeling tool of Rosetta and homology modeling, since templates with cover and identity percentages close to 100% were found. Successively, we performed aMD simulations on the different Fabs. Residue contact maps obtained from analysis of aMD trajectories showed the proximity of the CDR3 of the heavy and light chains for the unfunctional Fab-xiRB49. This contact is not present for functional Fab-RB49 and is almost completely lost for Fab-xiRB49-P125T, as also showed by Figure S8, where the CDRs solvent exposed surfaces are shown. These results indicate that the chimerization of Fab-RB49 induces a structural change that affects the relative conformations of the heavy and light chain CDR3, which might be responsible for the loss of the affinity of xiRB49 for ET_B_. Conversely, the P125T mutation in xiRB49 alters the xiRB49 inactive conformation, switching it to a structure closer to that of RB49. Indeed, the exact RB49 CDRs orientation is not recovered by xiRB49-P125T, since the medium frequency contacts (30–40% of the simulation time) between H-CDR2 and L-CDR3 are not restored by the mutation, and some residual contacts between the heavy and light chains CDR3 are still observable. This can be due to the long-range effects of the heavy chain constant region, since the RB49 and xiRB49 CH1 have an identity percentage of 65%, with 14 out of the 36 different residues being at the CH1 N-ter (Figure S7 in supplemental data). In addition, the simulations on Fab-xiRB49-P125T were started from the manually mutated Fab-xiRB49, and they share the same constant regions. Therefore, some of the Fab-xiRB49 contacts and structural features might persist during the simulations of Fab-xiRB49-P125T. Nevertheless, it is remarkable that the structural effects of a single point mutation are already visible in the generated trajectories.

To understand how P125 can have this long-range effect, we analyzed the simulations of the three systems by performing an analysis of the H-bond network around this position. We observed that Fab-RB49 is characterized by stable H-bonds connecting the heavy chain constant and variable regions, contributing to the relative orientation of the CDRs. Conversely, Fab-xiRB49 showed a significantly weakened H-bond network, probably because most of the sequence differences between the chosen mouse and human heavy chain constant regions are localized at their N-termini, as previously mentioned. For example, the H-bond between S96 and D188 cannot be established in xiRB49, since at this position there is a glycine instead of an aspartate (Figure S7 and HC-RB49-Fab sequence in supplemental data). Thus, P125, an amino acid known to have a relevant effect on peptide and protein structures, and the changes within the H-bond networks, might be responsible for the observed Fab-xiRB49 conformational change as compared to Fab-RB49, leading to the loss of the ET_B_ recognizing capabilities.

Threonine within Fab-xiRB49-P125T seems to reestablish a H-bond network between the amino acids surrounding the mutation and to reorient the CDRs. In addition, the presence of an additional H-bond network in Fab-xiRB49-P125T might also explain its T_i_2 increase as compared to xiRB49 T_i_2. Indeed, it is known that the thermostability of proteins is positively affected by weak interactions: the higher the number of stable interactions, the higher the melting temperature.^24^

To conclude, we have shown that the RB49 isotype switch was inefficient because of a single amino acid. It would be interesting to explore whether other antibodies have a proline in the considered position and lose their affinity during chimerization. If so, this mutation could be helpful for the chimerization of other therapeutic antibodies. Indeed, we show here that a single point mutation of P125 to threonine could be sufficient for successful chimerization. This important step paves the way for the humanization of xiRB49, which is required for the clinical development of immunotherapeutic applications of this antibody in oncology.

## Materials and methods

### Cell culture and antibody recombinant expression

Three established Chinese hamster ovary (CHO) cell lines were used: established with ET_A_: CHO-ET_A_, established with ET_B_: CHO-ET_B_, and wild type: CHO-WT. Cells were cultured as previously described.^10,11^ RB49-VH (variable heavy) and RB49-VL (variable light) were merged with the human IgG1/Kappa constant regions and expressed in ExpiCHO-S cells (Thermo Fisher Scientific, A29127). mAbs were purified on Protein A (GE Healthcare-17-0402-03). Sequences are indicated in SI.

### Fluorescent flow cytometry experiments

Binding experiments were performed using CHO cells expressing or not expressing endothelin receptors. Cells were incubated overnight at 4°C with antibodies ranging from 0.01 nM to 100 nM and detected with Alexa-Fluor 488^TM^ conjugated to goat anti-mouse IgG (Invitrogen-A10684) or F(ab’)2-goat anti-human IgG Fc (Invitrogen-H10120). Fluorescence intensities were quantified with the FACSCalibur flow cytometer (BD Biosciences, San Jose, USA) according to previously described protocols.^10,11^ Then, the mean fluorescence intensity (MFI) was determined by the cytometer software and the data were analyzed with GraphPad Prism software (v9.0.1). We normalized the MFI data, after checking that the data had a normal distribution, and fitted curves with the function “one site specific binding” allowing the determination of the apparent Kd and the Bmax. Student t-tests (two-tailed) were carried out to compare two data groups.

### Thermal denaturation experiments

Samples at 1 mg⋅mL^−1^ were deposited into the capillaries to determine the antibody denaturation temperature curves (Tycho NT6 instrument, NanoTemper). The ratio A350 nm/A330 nm was calculated, and its first derivative gave us an inflection temperature (T_i_) corresponding to the conformational change of the protein domain from the native to the denatured state.

### Dynamic light scattering

Samples at 1 mg/mL was dropped into the cuvette and measured with DynaPro NanoStar I (Wyatt technology). The software allowed us to confirm the absence of aggregates and to determine the hydrodynamic radius of our samples.

### Fabs *in silico* modeling

The variable regions of RB49, including the H-CDR3 loop, were modeled using the Antibody modeling tool implemented on the Rosie web-server.^25^ The obtained model quality was verified using MolProbity.^26^ To validate the results, the light and heavy chain variable regions were independently submitted to a BLAST search (https://blast.ncbi.nlm.nih.gov/Blast.cgi), using the PDB database to confirm the quality of the variable regions modeled by Rosie. We repeated the BLAST search for the constant regions. Since 100% identity was found for the light chain constant regions of both RB49 and xiRB49, we used the 1FIG and 6U8K kappa constant regions as they are for the mouse and chimeric RB49 modeling. For the heavy chain constant regions, we performed homology modeling with the SwissModel webserver using 1IGY and 1HZH as templates.^27^ The models were evaluated in terms of reliability of the model and geometrical features. The RB49 and xiRB49 Fabs were finally reconstructed by joining the variable and constant regions with PyMol.^28^ Further details are available as Supplemental Material. The xiRB49 P125T mutant was obtained by manually mutating the residue. Finally, the protonation states of the amino acids of the models were computed with the H++ server in physiological conditions.

### Molecular dynamic simulations on RB49 Fabs

The three modeled Fabs were submitted to classical molecular dynamics (cMD) simulations performed with the *pmemd.cuda* module of the Amber20 package^29^ using the ff14SB force field.^30^ The total charge was neutralized by including an adequate number of Na^+^/Cl^−^ ions and the systems were embedded in an octahedral TIP3P water box added up to 10 Å from the solute. Each system was then submitted to a cMD procedure detailed in the Supplementary Data. These simulations were used to collect average potentials for the following accelerated MD (aMD) simulations.

For RB49 and xiRB49 Fab, 3 independent aMD of 1 μs each were run, for a total of 3 μs. Conversely, in order to better consider the single point mutation, for xiRB49-P125T Fab, 3 independent aMD of 1.5 μs each were run, for a total of 4.5 μs. Cluster analyses were performed with the *cpptraj* Amber20 module using the average-linkage algorithm, requesting 10 clusters and setting the pairwise mass-weighted RMSD on the Cα backbone atoms as a metric. The same module was used to compute the distances between the centers of mass of the heavy and light chain CDRs and the inter- and intrachain H-bonds by requesting 3.5 Å and 130° as distance and angle cutoffs, respectively.

The R bio3D package was used to compute the contact maps on the aMD trajectories.^31^ The upper threshold to consider two atoms in contact was set to 12 Å. The contact maps were averaged over the 3 independent runs of each system.

## Supplementary Material

Supplemental Material
