# Supplementary material for: The functionality of a therapeutic antibody candidate restored by a single mutation from proline to threonine in the variable region
Source: Hum Vaccin Immunother. 2023 Nov 27;19(3):2279867. doi: 10.1080/21645515.2023.2279867 (PMC10760395; doi:10.1080/21645515.2023.2279867)
Supplement: Supplemental Material [file KHVI_A_2279867_SM9620.pdf]

MATERIALS AND METHODS SUPPLEMENTAL DATA

Expression of RB49, xi RB49 and xiRB49 P115T:

The rendomab B49, targeting ET_B receptor, was obtained by a novel gene immunization approach according to the laboratory protocol published in 2011. The originality of this approach is to present endothelin receptors in native conformations in an *in vivo* context.

Gene encoding the constant and variable sequences were synthesized by Eurofins Genomics Company. Then, sequences were subcloned into the pTT5 vector.[1]

Then, the vectors were added in 1 mL of chemically competent TOP10 bacteria transformed by a thermic shock (30 s at 42°C). Transformed bacteria were plated on LB agar with ampicillin and incubated at 37°C overnight. Plasmids were purified for sequencing using the Wizard® Plus SV Minipreps DNA Purification System (A1340).

Different constructions: RB49, xiRB49, xiRB49-P125T, Fab-xiRB49 and Fab-xiRB49-P125T were expressed in ExpiCHO-S cells according to the manufacturer's instructions (ThermoFisher Cat # A29127). We followed the instructions of the "Max Titer Protocol" with the addition of 300µL of ExpiFectamin™-CHO-Enhancer and 8mL of ExpiCHO™-feed on day 1 and day 5 post-transfection. Cells were shifted to 32°C on day 1 post transfection. To clarify the cell culture supernatant day 12 post transfection, we centrifuged it at 4000 g for 30 minutes at 4°C and filtered the supernatant through a 0.22µm filter.

We purified mAbs from culture medium on a HiTrap Protein A HP column (GE Healthcare, Cat.17-0402-03). Following elution, we dialysed the purified mAb in Slide-A-Lyzer® Dialysis Cassette (Thermoscientific 66030) into 1L of 1X PBS.

SDS-PAGE:

To assess the quality and the purification of the proteins, we analyzed samples using the 4-15%mini protean® precast TGX™ (Tris-Glycine eXtended) Gels (BIORAD #4561083). Samples were

treated in a different way, either with a non-reduced-buffer (Native Sample Buffer #1610738) either with a reduced-buffer (Laemmli 4X #1610747). Samples and molecular weight markers, Precision Plus Protein™ Markers (Biorad #1610363), were denatured at 95°C during 10 minutes and loaded on precast gels. Migrations were performed at a constant voltage of 200 V during 15 minutes.

Protein sequences

Fab-RB49

HC-RB49-Fab

QVQLQQPGAALVKPGASVKLSCKASGYTFISYWMLWVKQRPGRGLEWIGRIDPDSGGTKYNEKFKSKATLTVDKS

96 125

SSTAYMQLSSLTSEDSAVYYCAREGDYAWFAYWGQGTLV**P**VSAAKTTPPSVYPLAPGSAAQTNSMVTLGCLVKG

188

YFPEPVTVTWNSGSLSSGVHTFPAVLQSDLYTLSSSVTVPSSTWPSETVTCNVAHPASSTKVDKKI

LC-RB49

DVLMTQTPLSLPVSLGDQASISCRSSQSIVHSNGNTYLEWYLQKPGQSPKLLIYKVSNRFSGVPDRFSGSGSGTDFTL

KISRVEAEDLGVYYCFQGSHVPWTFGGGTKLEIKRADAAPTVSIFPPSSEQLTSGGASVVCFLNNFYPKDINVKWKI

DGSERQNGVLNSWTDQDSKDSTYSMSSTLTLTKDEYERHNSYTCEATHKTSTSPIVKSFNRNEC

Fab-xiRB49

HC-xiRB49-Fab

QVQLQQPGAALVKPGASVKLSCKASGYTFISYWMLWVKQRPGRGLEWIGRIDPDSGGTKYNEKFKSKATLTVDKS

SSTAYMQLSSLTSEDSAVYYCAREGDYAWFAYWGQGTLV**P**VSAASTKGPSVFPLAPSSKSTSGGTAALGCLVKDY

FPEPVTVSWNSGALTSGVHTFPAVLQSSGLYSLSSVVTVPSSSLGTQTYICNVNHKPSNTKVDKKV

LC-xiRB49

DVLMTQTPLSLPVSLGDQASISCRSSQSIVHSNGNTYLEWYLQKPGQSPKLLIYKVSNRFSGVPDRFSGSGSGTDFTL

KISRVEAEDLGVYYCFQGSHVPWTFGGGTKLEIKRTVAAPSVFIFPPSDEQLKSGTASVVCLLNNFYPREAKVQWKV

DNALQSGNSQESVTEQDSKDSTYSLSSTLTLSKADYEKHKVYACEVTHQGLSSPVTKSFNRGEC

Fab-xiRB49-P125T

HC-xiRB49-Fab

QVQLQQPGAALVKPGASVKLSCKASGYTFISYWMLWVKQRPGRGLEWIGRIDPDSGGTKYNEKFKSKATLTVDKS

SSTAYMQLSSLTSEDSAVYYCAREGDYAWFAYWGQGTLV**T**VSAASTKGPSVFPLAPSSKSTSGGTAALGCLVKDY

FPEPVTVSWNSGALTSGVHTFPAVLQSSGLYSLSSVVTVPSSSLGTQTYICNVNHKPSNTKVDKKV

LC-xiRB49

DVLMTQTPLSLPVSLGDQASISCRSSQSIVHSNGNTYLEWYLQKPGQSPKLLIYKVSNRFSGVPDRFSGSGSGTDFTL

KISRVEAEDLGVYYCFQGSHVPWTFGGGTKLEIKRTVAAPSVFIFPPSDEQLKSGTASVVCLLNNFYPREAKVQWKV

DNALQSGNSQESVTEQDSKDSTYSLSSTLTLSKADYEKHKVYACEVTHQGLSSPVTKSFNRGEC

RB49 Fabs modeling

The RB49 variable regions were modelled by the Antibody modeling tool of the Rosetta webserver, by asking to also model the H-CDR3. The obtained model was submitted to a quality check on the MolProbity webserver. Figure S9 shows the resulting report, which indicates a model of good quality (MolProbity score of 1.28 in the 99th percentile), without Ramachandran outliers and with a good clashscore. The only minor issues come from other geometrical features, such as bonds lengths (0.44% of bad bonds) and angles amplitudes (0.53% of bad angles). However, this kind of issues are resolved during the following classical molecular dynamics simulation. In addition, the light and heavy chain variable regions were independently submitted to a BLAST search (https://blast.ncbi.nlm.nih.gov/Blast.cgi), using the PDB database to confirm the quality of the variable regions modeled by Rosie. The BLAST search on the variable region of the heavy chain provided an alignment with a cover percentage of 100%, an identity percentage of 86.67%, and an E-value of $6{\cdot}10^{-10}$ with the variable region of the heavy chain of a Fv with code PDB 1A6U, while the one on the variable of the light chain provided an alignment with 100% of cover and identity percentages and an E-value of $1{\cdot}10^{-79}$ with the variable region of a Fab fragment with code PDB 4TPR. Figure S10 reports the superposition of the model to the two structures retrieved by the BLAST searches, which supports the conclusions on the quality of the variable regions model.

We repeated the BLAST search for the constant regions. For the RB49 light chain constant region we found an alignment with 100% of cover and identity percentages and an E-value of $3{\cdot}10^{-77}$ with the constant region of the light chain of the Fab with code PDB 1FIG. In addition, the variable regions of the light chains of RB49 and 1FIG show many conserved positions (Figure S11), thus we decided to use this structure to build the RB49 light chain constant region. The BLAST search performed on the xiRB49 kappa constant region gave an alignment with 100% of cover and identity percentages and an E-value of $1{\cdot}10^{-75}$ with the kappa constant region of a Fab with code PDB 6U8K, and the alignment on the whole xiRB49 and 6U8K light chain showed many conserved residues also in the variable region (Figure S12), making this structure exploitable for the xiRB49 light chain modeling.

Analogously, we performed a BLAST search on the full RB49 constant region, and we obtained an alignment with 100 % of cover, 94.38% of identity and an E-value of 0 with the constant region of the heavy chain of an IgG1 with code PDB 1IGY. In addition, the alignment with the whole heavy chain showed a high number of conserved residues in the variable region also (Figure S13). Therefore, we proceeded with the homology modeling of the RB49 CH1 using the 1IGY constant region CH1 with SwissModel. We obtained a model with a GMQE of 0.82 and an QMeanDisCo Global of 0.76 ± 0.09, indicating a model of good quality. The structural assessment provided by SwissModel indicates a model with a few issues, notably 4 Ramachandran outliers, 2 distorted bonds and 11 distorted angles. However, these geometrical issues are resolved during the classical molecular dynamics simulations.

The same procedure was applied on the xiRB49 heavy chain constant region. The BLAST search provided an alignment with the constant region of the heavy chain of an IgG (code PDB 1HZH) with 100% cover, 99.08% identity, and an E-value of 0. In addition, many conserved residues were found also in the variable region, as shown in the alignment of the xiRB49 heavy chain and 1HZH heavy chain of Figure S1. Therefore, we proceeded with the homology modeling of the xiRB49 heavy chain CH1 with SwissModel using the 1HZH heavy chain as template. In this case, we obtained a GMQE of 0.88 and a QMEANDisCo Global of 0.82 ± 0.08, suggesting a model of good quality. In addition, the SwissModel structure assessment results indicated the absence of Ramachandran outliers and of distorted bonds, while we found 9 distorted angles.

Finally, we built the final Fab-RB49 and Fab-xiRB49 by simultaneously aligning the modelled structures to the structures used as templates, manually creating a bond between the variable and constant regions, and finally minimizing the obtained structures with a minimization consisting in 2500 cycles of steepest descent and 5000 cycles of conjugated gradient.

Molecular dynamics simulations on RB49 Fabs

The modelled mouse, chimeric and mutated chimeric RB49 Fabs have been successively submitted to classical molecular dynamics (cMD) simulations. These were performed with the *pmemd.cuda* module of Amber20 package[2] using the ff14SB force field [3]. For each Fab, the total charge

was neutralized by including an adequate number of Na^+/Cl^- ions and the systems were embedded in an octahedral TIP3P water box added up to 10 Å from the solute. Each system was then relaxed by optimizing the hydrogens geometry (1000 cycles of steepest descent and 5000 cycles of conjugated gradient), ions and water molecules (2000 cycles of steepest descent and 5000 cycles of conjugated gradient). The water box was equilibrated at 300 K by 100 ps of NVT and 100 ps of NPT simulation using a Langevin thermostat with a collision frequency of 2.0 ps^{-1}. Then, we performed a minimization of side chains, water and ions by applying backbone restraints of 25 kcal/mol and a total minimization with backbone restraints of 10 kcal/mol (2500 cycles of steepest descent and 5000 cycles of conjugated gradient). Each system was then gradually brought to 300K in 6 steps of 5 ps each with an temperature increase step of 50 K, while backbone restraints were progressively reduced from 10 to 5 kcal/mol. A 100 ps NVT equilibration step followed by a 200 ps NPT equilibration step (backbone restraints = 5 kcal/mol) were performed. Successively, the backbone restraints were gradually removed by 100 ps NPT equilibration steps. Finally, 10 ns of unrestrained production at 300 K were performed to collect average potentials for the following accelerated MD (aMD) simulations. During the cMD simulations an electrostatic cutoff of 8.0 Å, a Berendsen barostat, PME for long-range electrostatic interactions and the SHAKE algorithm were applied.

Finally, for RB49 and xiRB49 Fabs 3 independent aMD runs of 1 μs each were run, for a total of 3 μs. Conversely, in order to better consider the single point mutation, for the p125t-xiRB49 Fab 3 independent aMD runs of 1.5 μs each were run, for a total of 4.5 μs.

SUPPLEMENTARY FIGURES AND TABLES

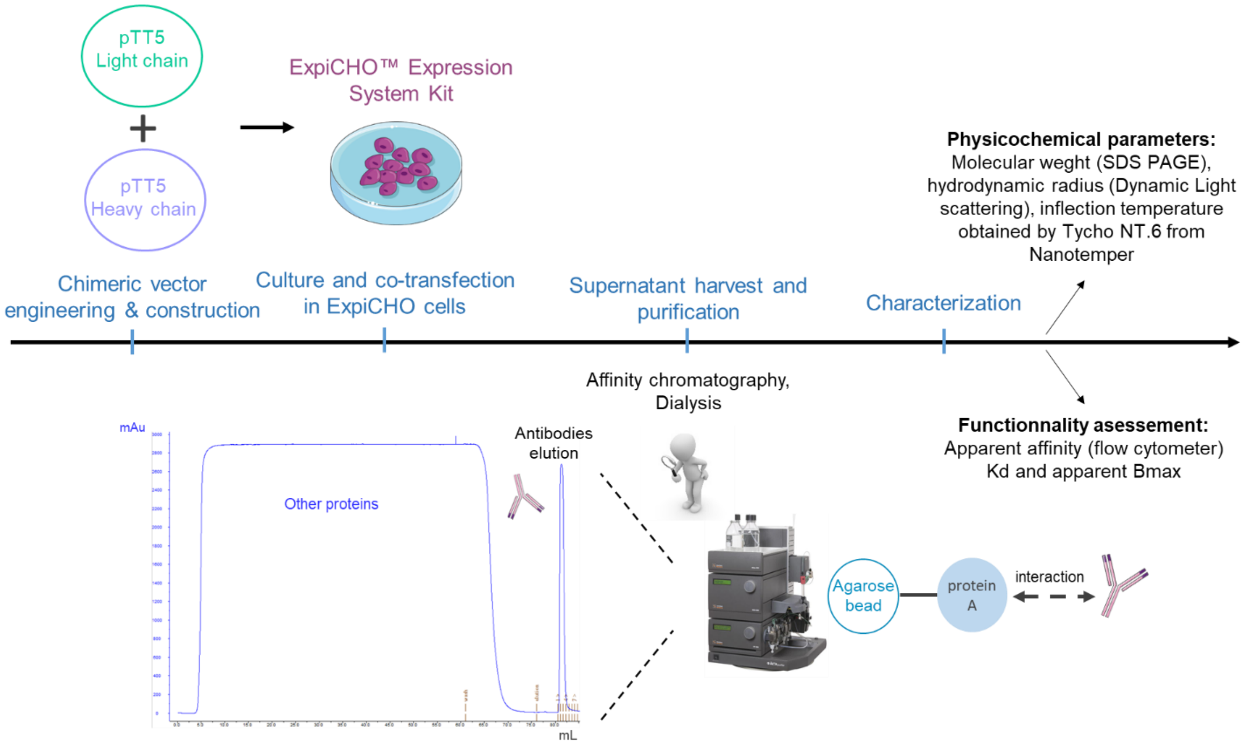

Figure S1. Main steps for chimerisation, production and quality controls performed to validate the functionality of our antibodies. To generate the xiRB49, the human constant light (Kappa) and heavy (IgG1) chain sequences were merged with the RB49 variable regions in the pTT5 expression vector. Plasmids were cotransfected into ExpiCHO-S cells. Day 12 post transfection, supernatant was clarified and filtered. The mAb was purified on Protein A column and dialyzed in PBS buffer. Purity and quality were assessed by SDS-PAGE, DLS, thermic denaturation curve and functionality by flow cytometry.

a.

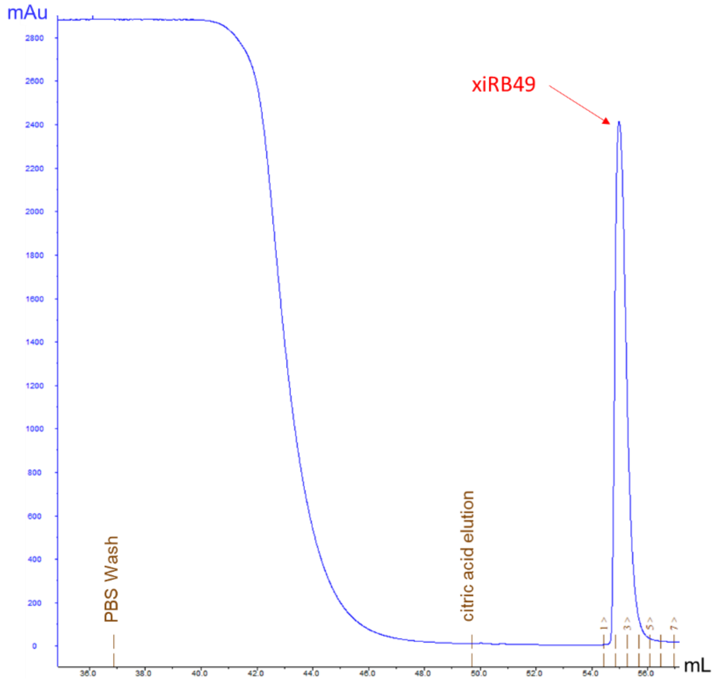

b.

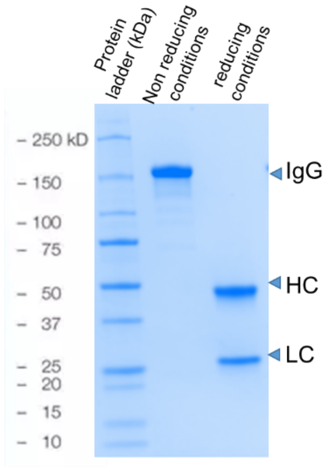

c.

	RB49	xiRB49	RA63	xiRA63
Radius (nm)	5.1±0.4	5.2±0.4	4.9±0.3	5.1±0.1

Figure S2. xiRB49 physicochemical parameters. (a) xiRB49 purification UV chromatogram (280nm) on A protein column. First signal corresponds to the whole nonspecific proteins before the PBS wash. The second peak corresponds to the xiRB49 antibody elution caused by citric acid. (B.) SDS PAGE of the xiRB49 in non reducing conditions heated at 95°C during 5 minutes (left) and in reduced condition by βmercaptoethanol (right). We used the 1kb plus protein ladder on the left of the figure to compare the different molecular weights. (C.) Hydrodynamic radius of the xiRB49, RB49, xiRA63 RA63 obtained by DLS (n=3). Data are presented as mean ± SD.

A.

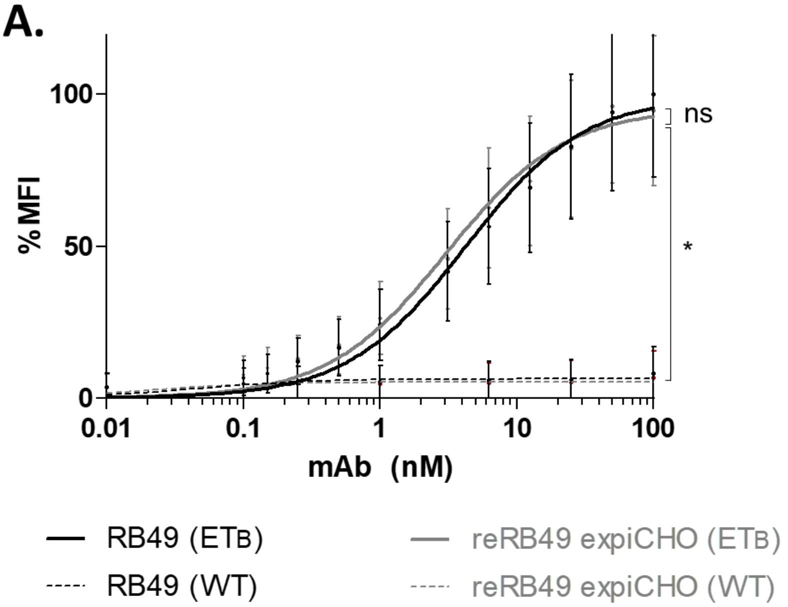

B.

	Rendomab B49	Recombinant RB49 (reRB49)
**Bmax (%MFI)**	99.7 ± 0.1	95.7 ± 0.1
**Kd (nM)**	4.19 ± 0.02	3.04 ± 0,02

Figure S3. FACS binding curves of RB49 and recombinant RB49**.** The binding curve of RB49 (black) and reRB39 (grey) are similar. Both mAbs were ET_B specific (solid line) as no signal was detected on CHO-WT (dotted line). Data presented as mean ± SD. MFI: Median Fluorescence Intensity.

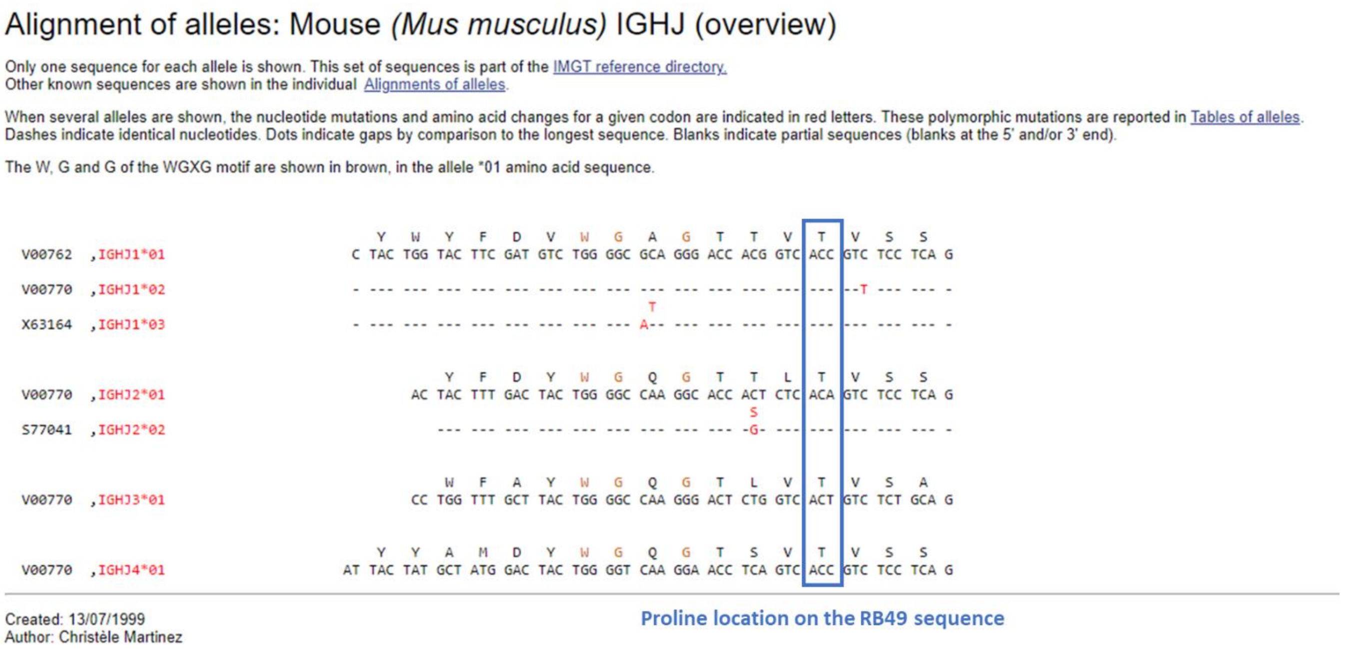

Figure S4. Screen capture of the mouse IGHJ alignment overview on the IMGT website (https://www.imgt.org/IMGTrepertoire/Proteins/alleles/index.php?species=Mus%20musculus&group=IGHJ&gene=IGHJ-overview). The IMGT/V-QUEST reference directory sets consist of sequences in FASTA format that include the V-REGION, D-REGION, and J-REGION alleles. These alleles are extracted from the Functional (F), ORF, and in-frame pseudogene (P) IMGT reference sequences. Each set contains a single sequence for each allele. Allele names for these sequences are in red in the alignments of alleles. The blue box shows the absence of genome-encoded Proline in mouse antibody FR4 and the constant presence of a Threonine.

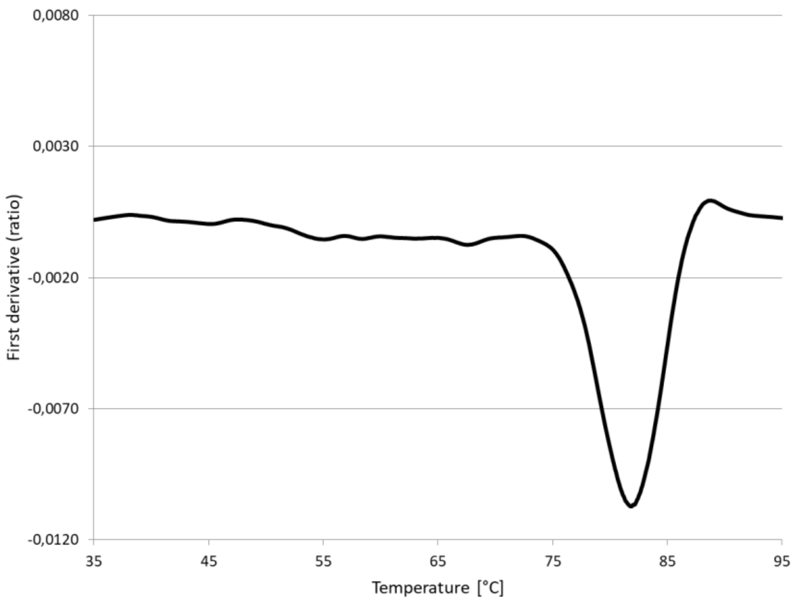

	RB49
Ti1 (full antibody)	81.8°C
Ti2	

Figure S5. Temperature curve of the Rendomab B49 obtained by Tycho NT.6: First derivated (ratio A350nm/A330nm) of the RB49 and its summary inflection temperature (Ti) table obtained by Tycho NT.6. Ti: inflection temperature

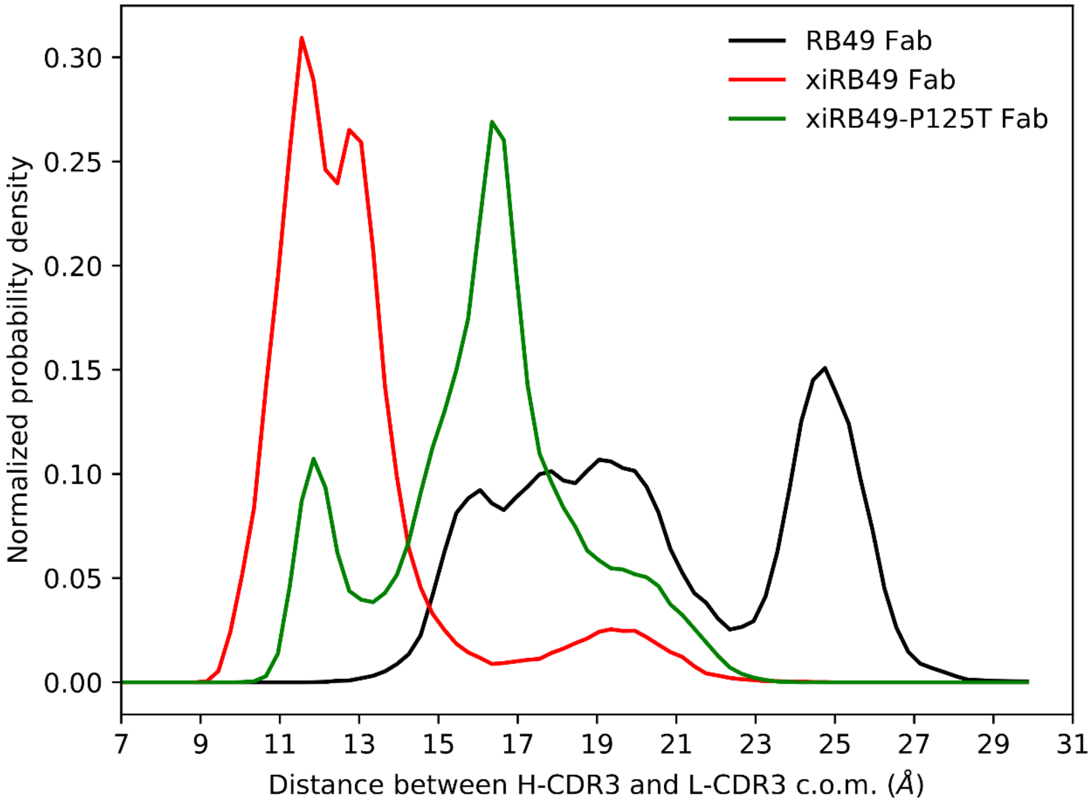

Figure S6. Probability distributions of the RB49 (black), xiRB49 (red) and xiRB49-P125T (green) H-CDR3 and L-CDR3 center of mass distances.

```
CLUSTAL O(1.2.4) multiple sequence alignment


Fab-RB49 heavy chain        QVQLQQPGAALVKPGASVKLSCKASGYTFISYWMLWVKQRPGRGLEWIGRIDPDSGGTKY    60
Fab-xiRB49 heavy chain      QVQLQQPGAALVKPGASVKLSCKASGYTFISYWMLWVKQRPGRGLEWIGRIDPDSGGTKY    60
                            ************************************************************

Fab-RB49 heavy chain        NEKFKSKATLTVDKSSSTAYMQLSSLTSEDSAVYYCAREGDYAWFAYWGQGTLVPVSAAK    120
Fab-xiRB49 heavy chain      NEKFKSKATLTVDKSSSTAYMQLSSLTSEDSAVYYCAREGDYAWFAYWGQGTLVPVSAAS    120
                            ***********************************************************.

Fab-RB49 heavy chain        TTPPSVYPLAPGSAAQTNSMVTLGCLVKGYFPEPVTVTWNSGSLSSGVHTFPAVLQS-DL    179
Fab-xiRB49 heavy chain      TKGPSVFPLAPSSKSTSGGTAALGCLVKDYFPEPVTVSWNSGALTSGVHTFPAVLQSSGL    180
                            *. ***:****.* : :.. .:******.********:****:*:************ .*

Fab-RB49 heavy chain        YTLSSSVTVPSSTWPSQTVTCNVAHPASSTKVDKKIVPRDC    220
Fab-xiRB49 heavy chain      YSLSSVVTVPSSSLGTQTYICNVNHKPSNTKVDKKVEPKSC    221
                            *:*** ******:  :**  *** *  *.******: *:.*
```

Figure S7. Sequence alignment of the Fab-RB49 and Fab-xiRB49 heavy chains. The alignment has been obtained by Clustal Omega 1.2.4. * indicate identical amino acids, highly conserved amino acids and poorly conserved amino acids. The amino acid difference, G to D in the constant region, red box, is numbered 178 in classical numbering and 188 in IMGT numbering.

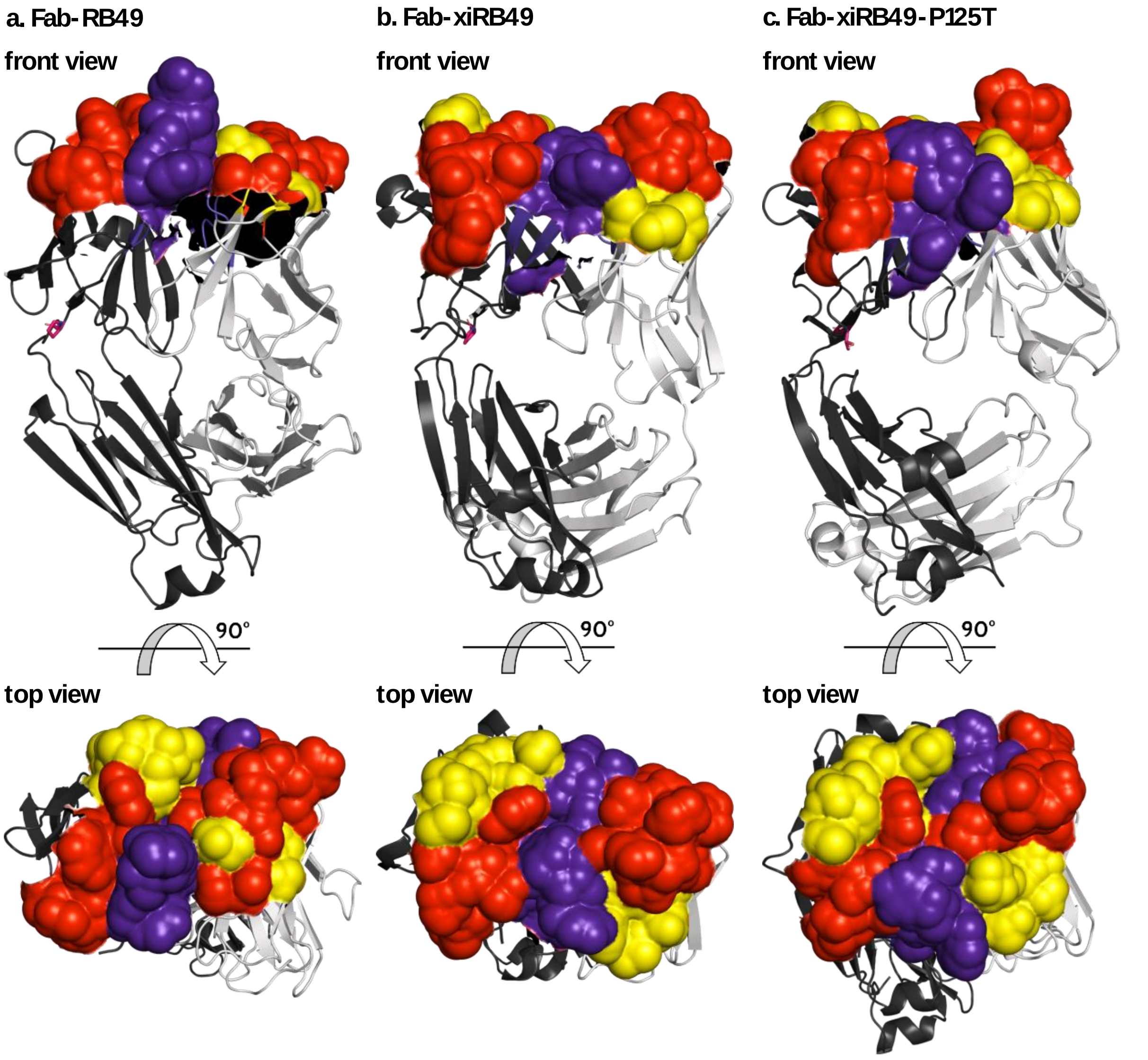

Figure S8. Representative structures of the most populated cluster of (a) Fab-RB49, (b) Fab-xiRB49, and (c) Fab-xiRB49-P125T. The heavy chain and the light chain are represented in dark and light grey, respectively. CDR1, CDR2, and CDR3 solvent exposes surfaced are colored red, yellow, and purple, respectively. The residue in position 125 (either proline or threonine) is represented as ball and sticks and colored magenta.

Summary statistics

All-Atom Contacts	Clashscore, all atoms:	4.55		95th percentile* (N=1784, all resolutions)
	Clashscore is the number of serious steric overlaps (> 0.4 Å) per 1000 atoms.			
Protein Geometry	Poor rotamers	1	0.52%	Goal: <0.3%
	Favored rotamers	192	98.97%	Goal: >98%
	Ramachandran outliers	0	0.00%	Goal: <0.05%
	Ramachandran favored	220	97.78%	Goal: >98%
	Rama distribution Z-score	-0.62 ± 0.53		Goal: abs(Z score) < 2
	MolProbity score^	1.28		99th percentile* (N=27675, 0Å - 99Å)
	Cβ deviations >0.25Å	0	0.00%	Goal: 0
	Bad bonds:	8 / 1825	0.44%	Goal: 0%
	Bad angles:	13 / 2475	0.53%	Goal: <0.1%
Peptide Omegas	Cis Prolines:	2 / 11	18.18%	Expected: ≤1 per chain, or ≤5%
Low-resolution Criteria	CaBLAM outliers	1	0.5%	Goal: <1.0%
	CA Geometry outliers	1	0.45%	Goal: <0.5%
Additional validations	Chiral volume outliers	0/264		
	Waters with clashes	0/0	0.00%	See UnDowser table for details

Figure S9. RB49 variable regions model quality assessment by Molprobity. * 100th percentile is the best among structures of comparable resolution; 0th percentile is the worst. For clashscore the comparative set of structure was selected in 2004, for MolProbity score in 2006. ^ Molprobity score combines the clashscore, rotamer, and Ramachandran evaluations into a single score, normalized to be on the same scale as X-ray resolution.

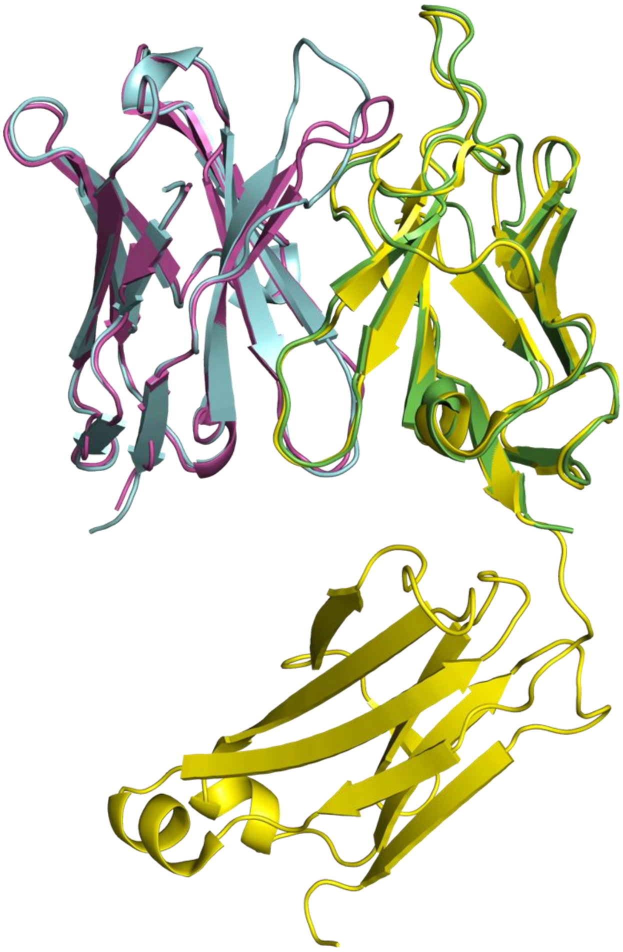

Figure S10. Superposition of the modelled RB49 variable regions with the structures selected after the BLAST searches. The RB49 heavy chain variable region (magenta) is superposed to the heavy chain

variable region of a Fv with code PDB 1A6U (cyan), while the RB49 light chain variable region (green) is superposed to the light chain of a Fab with code PDB 4TPR (yellow).

```
CLUSTAL O(1.2.4) multiple sequence alignment


1FIG_1|      ENVLTQSPAIMSASPGEKVTMACRASSSV----SSTYLHWYQQKSGASPKLLIYSTSNLA      56
RB49         DVLMTQTPLSLPVSLGDQASISCRSSQSIVHSNGNTYLEWYLQKPGQSPKLLIYKVSNRF      60
             : ::**:*  : .* *::.:::**:*.*:    ..***.** ** * *******..**

1FIG_1|      SGVPARFSGSGSGTSYSLTISSVEAEDAATYYCQQYSGYPLTFGAGTKLELKRADAAPTV      116
RB49         SGVPDRFSGSGSGTDFTLKISRVEAEDLGVYYCFQGSHVPWTFGGGTKLEIKRADAAPTV      120
             **** *********.::*.** ***** ..*** * *  * ***.*****:*********

1FIG_1|      SIFPPSSEQLTSGGASVVCFLNNFYPKDINVKWKIDGSERQNGVLNSWTDQDSKDSTYSM      176
RB49         SIFPPSSEQLTSGGASVVCFLNNFYPKDINVKWKIDGSERQNGVLNSWTDQDSKDSTYSM      180
             ************************************************************

1FIG_1|      SSTLTLTKDEYERHNSYTCEATHKTSTSPIVKSFNRNEC    215
RB49         SSTLTLTKDEYERHNSYTCEATHKTSTSPIVKSFNRNEC    219
             ***************************************
```

Figure S11. Sequence alignment between the RB49 and the 1FIG light chains. The alignment has been obtained by Clustal Omega 1.2.4. * indicates identical amino acids, : highly conserved amino acids and . poorly conserved amino acids.

```
CLUSTAL O(1.2.4) multiple sequence alignment


6U8K_3|Chains      SDIQMTQSPSSLSASVGDRVTITCRASQSVS-----SAVAWYQQKPGKAPKLLIYSASSL 55
LC-xiRB49          -DVLMTQTPLSLPVSLGDQASISCRSSQSIVHSNGNTYLEWYLQKPGQSPKLLIYKVSNR 59
                    *: ***:* ** .*:**:.:*:**:***:      : : ** ****::******..*.

6U8K_3|Chains      YSGVPSRFSGSRSGTDFTLTISSLQPEDFATYYCQQYSSSPYTFGQGTKVEIKRTVAAPS 115
LC-xiRB49          FSGVPDRFSGSGSGTDFTLKISRVEAEDLGVYYCFQGSHVPWTFGGGTKLEIKRTVAAPS 119
                   :****.***** *******.** :: **:..*** * *  *:*** ***:**********

6U8K_3|Chains      VFIFPPSDEQLKSGTASVVCLLNNFYPREAKVQWKVDNALQSGNSQESVTEQDSKDSTYS 175
LC-xiRB49          VFIFPPSDEQLKSGTASVVCLLNNFYPREAKVQWKVDNALQSGNSQESVTEQDSKDSTYS 179
                   ************************************************************

6U8K_3|Chains      LSSTLTLSKADYEKHKVYACEVTHQGLSSPVTKSFNRGEC     215
LC-xiRB49          LSSTLTLSKADYEKHKVYACEVTHQGLSSPVTKSFNRGEC     219
                   ****************************************
```

Figure S12. Sequence alignment between the xiRB49 and the 6U8K light chains. The alignment has been obtained by Clustal Omega 1.2.4. * indicates identical amino acids, : highly conserved amino acids and . poorly conserved amino acids

```
CLUSTAL O(1.2.4) multiple sequence alignment

1IGY_2|heavy      -VKLQESGAELARPGASVKMSCKASGYTFTTYTIHWIKQRPGQGLEWIGYINPSSVYTNY  59
RB49              QVQLQQPGAALVKPGASVKLSCKASGYTFISYWMLWVKQRPGRGLEWIGRIDPDSGGTKY  60
                   *:**: ** *.:******:********* :* : *:*****:****** *:*.*  *:*

1IGY_2|heavy      NQRFKDKATLTRDRSSNTANIHLSSLTSDDSAVYYCVREGEV---PYWGQGTTVTVSSAK  116
RB49              NEKFKSKATLTVDKSSSTAYMQLSSLTSEDSAVYYCAREGDYAWFAYWGQGTLVPVSAAK  120
                  *::**.***** *:**.** ::******:*******.***:     ****** * **:**

1IGY_2|heavy      TTPPSVYPLAPGSAAQTNSMVTLGCLVKGYFPEPVTVTWNSGSLSSGVHTFPAVLQSDLY  176
RB49              TTPPSVYPLAPGSAAQTNSMVTLGCLVKGYFPEPVTVTWNSGSLSSGVHTFPAVLQSDLY  180
                  ************************************************************

1IGY_2|heavy      TLSSSVTVPSSPRPSETVTCNVAHPASSTKVDKKIVPRDCGCKPCICTVPEVSSVFIFPP  236
RB49              TLSSSVTVPSSTWPSQTVTCNVAHPASSTKVDKKIVPRDCGCKPCICTVPEVSSVFIFPP  240
                  ***********  **:********************************************

1IGY_2|heavy      KPKDTLLITVTPKVTCVVVDISKDDPEVQFSWFVDNVEVHTAQTQPREEQFNSTFRVVSA  296
RB49              KPKDVLTITLTPKVTCVVVDISKDDPEVQFSWFVDDVEVHTAQTKPREEQINSTFRSVSE  300
                  ****.* **:*************************:********:*****:***** **

1IGY_2|heavy      LPIMHQDWLNGKEFKCRVNSAAFPAPIEKTISKTKGKPRAPQVYTIPPPKEQMAKDKVSL  356
RB49              LPIMHQDWLNGKEFKCRVNSAAFPAPIEKTISKTKGRPKAPQVYTIPPPKEQMAKDKVSL  360
                  ************************************:*:*********************

1IGY_2|heavy      TCMITDFFPEDITVEWQSDGQAPENYKNTQPIMDTDGSYFVYSKLNVQKSNWEAGNTFTC  416
RB49              TCMITNFFPEDITVEWQWNGQPAENYKNTQPIMDTDGSYFVYSKLNVQKSNWEAGNTFTC  420
                  *****:*********** :**  *************************************

1IGY_2|heavy      SVLHEGLHNHHTEKSLSH    434
RB49              SVLHEGLHNHHTEKSLSH    438
                  ******************
```

Figure S13. Sequence alignment between the RB49 and the 1IGY heavy chains. The alignment has been obtained by Clustal Omega 1.2.4. * indicates identical amino acids, : highly conserved amino acids and . poorly conserved amino acids

```
CLUSTAL O(1.2.4) multiple sequence alignment

1HZH_1|heavy      QVQLVQSGAEVKKPGASVKVSCQASGYRFSNFVIHWVRQAPGQRFEWMGWINPYNGNKEF  60
xiRB49            QVQLQQPGAALVKPGASVKLSCKASGYTFISYWMLWVKQRPGRGLEWIGRIDPDSGGTKY  60
                  **** * ** : *******:**:**** * .: : **:* **: :**:* *:* .*..::

1HZH_1|heavy      SAKFQDRVTFTADTSANTAYMELRSLRSADTAVYYCARVGPYSWDDSPQDNYYMDVWGKG  120
xiRB49            NEKFKSKATLTVDKSSSTAYMQLSSLTSEDSAVYYCAREGDYAWF---------AYWGQG  111
                  . **:.:.*:*.*.*:.****:* ** * *:******* * *:*            **:*

1HZH_1|heavy      TTVIVSSASTKGPSVFPLAPSSKSTSGGTAALGCLVKDYFPEPVTVSWNSGALTSGVHTF  180
xiRB49            TLVPVSAASTKGPSVFPLAPSSKSTSGGTAALGCLVKDYFPEPVTVSWNSGALTSGVHTF  171
                  * * **:*****************************************************

1HZH_1|heavy      PAVLQSSGLYSLSSVVTVPSSSLGTQTYICNVNHKPSNTKVDKKAEPKSCDKTHTCPPCP  240
xiRB49            PAVLQSSGLYSLSSVVTVPSSSLGTQTYICNVNHKPSNTKVDKKVEPKSCDKTHTCPPCP  231
                  ********************************************.***************

1HZH_1|heavy      APELLGGPSVFLFPPKPKDTLMISRTPEVTCVVVDVSHEDPEVKFNWYVDGVEVHNAKTK  300
xiRB49            APELLGGPSVFLFPPKPKDTLMISRTPEVTCVVVDVSHEDPEVKFNWYVDGVEVHNAKTK  291
                  ************************************************************

1HZH_1|heavy      PREEQYNSTYRVVSVLTVLHQDWLNGKEYKCKVSNKALPAPIEKTISKAKGQPREPQVYT  360
xiRB49            PREEQYNSTYRVVSVLTVLHQDWLNGKEYKCKVSNKALPAPIEKTISKAKGQPREPQVYT  351
                  ************************************************************

1HZH_1|heavy      LPPSRDELTKNQVSLTCLVKGFYPSDIAVEWESNGQPENNYKTTPPVLDSDGSFFLYSKL  420
xiRB49            LPPSREEMTKNQVSLTCLVKGFYPSDIAVEWESNGQPENNYKTTPPVLDSDGSFFLYSKL  411
                  *****:*:****************************************************

1HZH_1|heavy      TVDKSRWQQGNVFSCSVMHEALHNHYTQKSLSLSPGK 457
xiRB49            TVDKSRWQQGNVFSCSVMHEALHNHYTQKSLSLS--- 445
                  *********************************
```

Figure S14. Sequence alignment between the xiRB49 and the 1HZH heavy chains. The alignment has been obtained by Clustal Omega 1.2.4. * indicates identical amino acids, : highly conserved amino acids and . poorly conserved amino acids

Table S1. Fab-RB49 hydrogen bonds occupancies.

Residue No.	Residue No.	Occ % (run 1)	Occ % (run 2)	Occ % (run 3)
S96/R45 (heavy chain)	D188 (heavy chain)	72.2	76.4	100
A128 (heavy chain)	T130 (heavy chain)	44.9	38.0	56.8
S127 (heavy chain)	A129/K130 (heavy chain)	35.4	24.0	27.7
E107 (heavy chain)	E40 (light chain)	60.0	58.6	nd
D109 (heavy chain)	K56 (light chain)	41.2	24.3	nd

Table S2. Fab-xiRB49 hydrogen bonds occupancies.

Residue No.	Residue No.	Occ % (run 1)	Occ % (run 2)	Occ % (run 3)
V13 (heavy chain)	S208 (heavy chain)	24.8	nd	nd
S127 (heavy chain)	A129 (heavy chain)	23.4	29.9	20.7
E107 (heavy chain)	W116 (light chain)	62.0	49.7	51.7

Table S3. Fab-xiRB49-P125T

Residue No.	Residue No.	Occ % (run 1)	Occ % (run 2)	Occ % (run 3)
S127 (heavy chain)	A129 (heavy chain)	68.3	72.9	70.8
A11 (heavy chain)	T125 (heavy chain)	97.0	97.4	97.3
V13 (heavy chain)	T125 (heavy chain)	93.8	nd	nd
E107 (heavy chain)	W116 (light chain)	65.1	59.2	nd
D109 (heavy chain)	Y55 (light chain)	31.7	nd	nd
D109 (heavy chain)	K56 (light chain)	29.8	nd	nd
E107 (heavy chain)	Y38 (light chain)	nd	nd	38.5
R106 (heavy chain)	E40 (light chain)	50.0	86.3	nd

REFERENCE Supplemental data

1. Durocher Y, Perret S, Kamen A. High-level and high-throughput recombinant protein production by transient transfection of suspension-growing human 293-EBNA1 cells. *Nucleic Acids Res*. 2002;30(2):E9. https://academic.oup.com/nar/article/30/2/e9/2380467?login=true

2. D.A. Case, K. Belfon, I.Y. Ben-Shalom, S.R. Brozell, D.S. Cerutti, T.E. Cheatham, III, V.W.D. Cruzeiro, T.A. Darden, R.E. Duke, G. Giambasu, M.K. Gilson, H. Gohlke, A.W. Goetz,R Harris, S. Izadi, S.A. Iz- mailov, K. Kasavajhala, A. Kovalenko, R. Krasny, T. Kurtzman, T.S. Lee, S. LeGrand, P. Li, C. Lin, J. Liu, T. Luchko, R. Luo, V. Man, K.M. Merz, Y. Miao, O. Mikhailovskii, G. Monard, H. Nguyen, A. Onufriev, F. Pan, S. Pantano, R. Qi, D.R. Roe, A. Roitberg, C. Sagui, S. Schott-Verdugo, J. Shen, C.L. Simmerling, N.R. Skrynnikov, J. Smith, J. Swails, R.C. Walker, J. Wang, L. Wilson, R.M. Wolf, X. Wu, Y. Xiong, Y. Xue, D.M. York and P.A. Kollman (2020), AMBER 2020, University of California, San Francisco.

3. Maier JA, Martinez C, Kasavajhala K, Wickstrom L, Hauser KE, Simmerling C. ff14SB: Improving the Accuracy of Protein Side Chain and Backbone Parameters from ff99SB. *J Chem Theory Comput*. 2015;11(8):3696-3713. https://pubs.acs.org/doi/10.1021/acs.jctc.5b00255
